# Inward lithium-ion breathing of hierarchically porous silicon anodes

**DOI:** 10.1038/ncomms9844

**Published:** 2015-11-05

**Authors:** Qiangfeng Xiao, Meng Gu, Hui Yang, Bing Li, Cunman Zhang, Yang Liu, Fang Liu, Fang Dai, Li Yang, Zhongyi Liu, Xingcheng Xiao, Gao Liu, Peng Zhao, Sulin Zhang, Chongmin Wang, Yunfeng Lu, Mei Cai

**Affiliations:** 1 Gereral Motors Research and Development Center, 30500 Mound Road, Warren, Michigan 48090, USA; 2 Environmental Molecular Sciences Laboratory, Pacific Northwest National Laboratory, Richland, Washington 99352, USA; 3 Department of Engineering Science & Mechanics, Pennsylvania State University, University Park, Pennsylvania 16802, USA; 4 Clean Energy Automotive Engineering Center, Tongji University, Shanghai 201804, China; 5 Department of Chemical and Biomolecular Engineering, The University of California, Los Angeles, California 90095, USA; 6 Environmental Energy Technologies Division, Lawrence Berkeley National Laboratory, Berkeley, California 94720, USA

## Abstract

Silicon has been identified as a highly promising anode for next-generation lithium-ion batteries (LIBs). The key challenge for Si anodes is large volume change during the lithiation/delithiation cycle that results in chemomechanical degradation and subsequent rapid capacity fading. Here we report a novel fabrication method for hierarchically porous Si nanospheres (hp-SiNSs), which consist of a porous shell and a hollow core. On charge/discharge cycling, the hp-SiNSs accommodate the volume change through reversible inward Li breathing with negligible particle-level outward expansion. Our mechanics analysis revealed that such inward expansion is enabled by the much stiffer lithiated layer than the unlithiated porous layer. LIBs assembled with the hp-SiNSs exhibit high capacity, high power and long cycle life, which is superior to the current commercial Si-based anode materials. The low-cost synthesis approach provides a new avenue for the rational design of hierarchically porous structures with unique materials properties.

## Abstract (web summary)

Porous silicon is highly promising as an anode material in lithium ion batteries, but its volume change upon lithiation has deleterious effects upon efficiency. Here, the authors demonstrate porous shell/hollow core Si nanospheres which exhibit negligible outward expansion and maintain performance.

## Graphical abstract

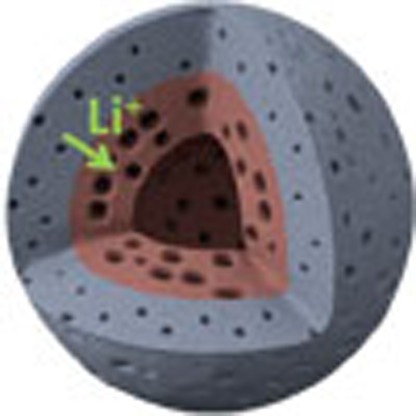

Lithium-ion batteries (LIBs) have emerged as the main power sources for microelectronics and are considered as the technology of choice for the vehicle electrification. Similar to other batteries, LIBs involve phase transformation accompanied by ion and electron transport. Maintaining effective and robust transport pathways, as well as minimizing reactions between electrodes and electrolytes, is the key to ensure batteries with high power and long cycling life. Silicon (Si), with a theoretical capacity of 4,200 mAh g^−1^, has been identified as one of the most promising anode candidates for the next-generation high-energy-density LIBs. However, Si anodes generally exhibit significant volume change during electrochemical cycling, resulting in pulverization of the particles, loss of the electrical contact, rupture of the solid-electrolyte interphase (SEI)^1^,^2^,^3^, and consequently, rapidly deteriorated storage performance.

Various strategies have been explored to mitigate these limitations, mainly by structural engineering of silicon particles, compositing with carbon (C) and adapting suitable binders. It has been demonstrated theoretically that Si materials in nanometre range exhibit alleviated lithiation-induced mechanical stress and enhanced resilience to fracture and decrepitation, which in turn result in enhanced ionic and electrical conductivities, and electrochemical performance^4^,^5^. Various Si materials with low-dimensional structures (for example, nanoparticles^6^,^7^, hollow spheres^8^, nest-like Si nanospheres^9^, nanowires^10^ and nanotubes^11^) or porous structures^12^ have been fabricated by mechanical ball milling, laser pyrolysis, chemical vapour deposition, solvothermal method and two-step thermal annealing/acid etching process. These materials have shown improved cycle life, capacity retention and rate performance. Similarly, the Si/C composites, wherein the carbon moieties serve as a cushion for the volume change and provide effective electronic pathways, also exhibit enhanced electrochemical performance compared with their pure silicon counterparts^13^,^14^,^15^. In the aspect of binders, sodium alginate^16^, poly(acrylic acid)^17^, conductive polymers^18^ and self-healing polymers^2^ have been employed to improve the inter-particle interactions, maintain or repair the electronic contacts during the cycling. However, the outward volume expansion of the above Si materials during lithiation and potential side reactions between binders and electrolytes are detrimental to prolonged cycling. Therefore, fabrication of high-performance Si anodes with minimal outward volume expansion remains challenging.

Herein we report a size-dependent chemical transformation method to fabricate hierarchically porous Si nanospheres (hp-SiNSs), which uniquely accommodate the volume change through reversible inward Li breathing on charge/discharge cycling.

## Results

### Synthesis of hp-SiNSs

**Figure 1:**
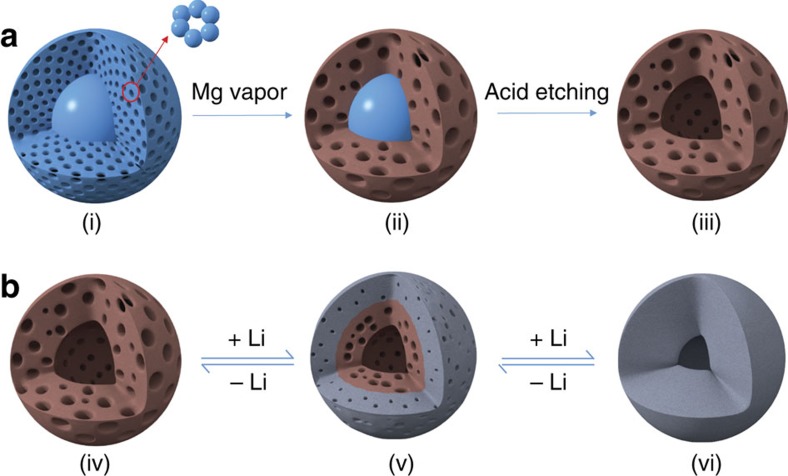
Schematic of synthesis method and lithiation/delithiation process for hp-SiNSs. (**a**) Schematic of synthesis of hp-SiNSs via size-dependent reduction. The method involves three steps: (i) synthesis of solid core/mesoporous shell SiO_2_ spheres by simultaneous hydrolysation and condensation of tetraethoxysilane and octadecyltrimethoxysilane, followed by the removal of organic species; (ii) conversion of the primary SiO_2_ nanoparticles mesoporous shell to Si nanoparticles by Mg vapour due to size-dependent reaction; (iii) acid etching to remove residual MgO and the solid SiO_2_ core to obtain the final hp-SiNSs with a mesoporous shell and hollow core. (**b**) Schematic of lithiation/delithiation process of the hp-SiNSs showing that the mesoporous shell directs the volume expansion towards the inner hollow core during the lithiation and recovers the morphology during the delithiation.

As illustrated in Fig. 1a, we started from silica (SiO_2_) spheres with a mesoporous shell and solid core. The SiO_2_ spheres were produced by simultaneous hydrolysation and condensation of tetraethoxysilane and octadecyltrimethoxysilane, followed by the removal of the organic species^19^. The shell can be visualized to be composed of nanoparticles of ∼3 nm in diameter, whereas the solid core has a diameter of hundreds of nanometres, as shown in Fig. 1a (i; step I). In the presence of Mg vapour, the SiO_2_ nanoparticles in the porous shell were converted to Si, while the solid SiO_2_ core remained nearly intact owing to the size-dependent transformation rate (step II). It's noteworthy to point out that possible structure reorganization in the shell can occur during this step, as indicated by the broader pore size distribution in Fig. 1a (ii) than that in Fig. 1a (i). Finally, hp-SiNSs were obtained by removing MgO (Supplementary Fig. 1a) and residual SiO_2_ through acid etching (step III). As schematically shown in Fig. 1b, the mesoporous shell directs the volume expansion towards the inner hollow core during the lithiation, which is enabled by the much stiffer lithiated layer than the unlithiated porous layer. During the delithiation, the morphology recovers. Opposite to the outward expansion in solid Si during lithiation, such inward Li breathing prevents the hp-SiNSs from fracture, and maintains stable SEI and ion and electron diffusion pathways. The resulting Si anodes are thus expected to possess high capacity, high power, long cycle life and high Coulombic efficiency. Previous studies demonstrated that double-walled Si nanotubes exhibit similar inward expansion and stable SEI formation, and hence impressively long cycle life^20^. However, the high cost and incompatibility with the current slurry coating electrode fabrication process prevent the nanotubes from commercialization.

### Characterization of hp-SiNSs

**Figure 2:**
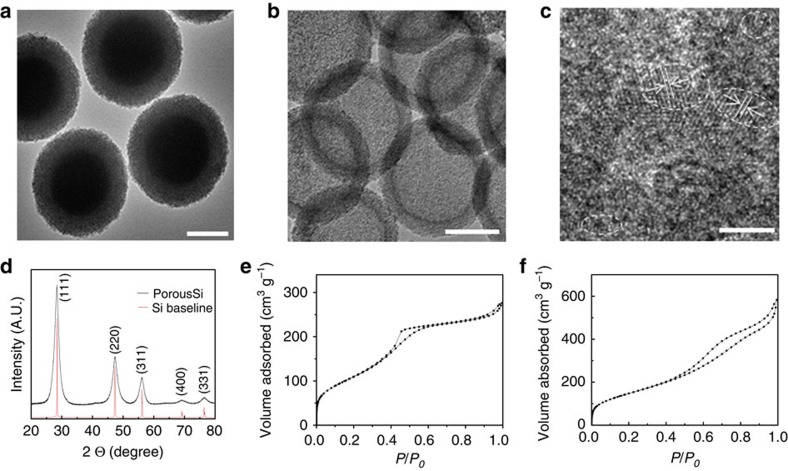
Structure characterizaion of the hp-SiNSs. (**a**) A TEM image of solid core/mesoporous shell SiO_2_ particles. (**b**) A low magnification TEM image and (**c**) a high-resolution TEM image of hp-SiNSs, showing the Si particles are mostly amorphous, with scattered nanocrystalline domains with a (111) interplanar spacing of 3.13 Å. (**d**) X-ray diffraction patterns of the hp-SiNSs. (**e**,**f**) N_2_ isotherms for solid core/mesoporous shell SiO_2_ particles and the hp-SiNSs, respectively. Scale bar, 200 nm (**a**,**b**) 4 nm (**c**).

Figure 2a shows the transmission electron microscope (TEM) images of monodisperse core/mesoporous shell SiO_2_ spheres with a shell thickness of 75 nm and a solid core of 350 nm in diameter. After the transformation, hp-SiNSs with a mesoporous shell and hollow core were obtained, as indicated by the contrast between the darker peripheral and the lighter central regions in Fig. 2b. A high-resolution TEM image indicates that the porous shell is mostly amorphous, scattered with only a few nanocrystalline domains with a (111) interplanar spacing of 3.13 Å (Fig. 2c)^21^. The hollow core was further confirmed by the scanning TEM image in the high-angle annular dark field imaging mode for which the image contrast is proportional to mass thickness and the square of the atomic number of the element. As shown in Supplementary Fig. 2, the outer layer of each hp-SiNS is much denser than its center. Furthermore, the mesoporous shell is demonstrated by homogeneously distributed black and white spots. In a control experiment, Si products could not be obtained when solid SiO_2_ spheres with the diameter of 350 nm were treated with the identical transformation steps (Supplementary Fig. 3). This further indicates that the size of SiO_2_ particles dictates the reaction rate. It is worthy to point out that similar hierarchically porous structures were obtained when changing shell thickness and core size of SiO_2_ (Supplementary Fig. 4).

Energy dispersive X-ray spectroscopy analysis showed the expected primary Si signal with a trace amount of oxygen, while no Mg signal was observed. As excessive HF solution was used to etch inner SiO_2_ core and the large-surface-area hp-SiNSs have high reactivity with O, we attribute the O to the air exposure during handling the sample. The Cu signal can be ascribed to TEM copper grid (Supplementary Fig. 1b). As quantified, The hp-SiNSs were constituted of 91 wt% Si and 9 wt% O. Consistently, X-ray diffraction exhibited characteristics of diamond cubic phase of Si (JCPDS card no. 27–1,402; Fig. 2d)^22^. The size of the crystallites was estimated to be 4.8 nm by the Scherrer equation. These results imply limited structure reorganization in the course of chemical transformation from silica precursor to the Si product. Raman spectrum was further used to verify the success of the conversion (Supplementary Fig. 1c). As compared with that of Si wafer reference (520 cm^−1^), the peak of the hp-SiNSs shifted to 508 cm^−1^. This shift was likely caused by the phonon confinement effect from the primary nanoparticles, whose first-order scattering peak shifts towards a lower energy as particle size decreases^23^,^24^.

Nitrogen adsorption–desorption isotherms show that the structures are preserved on the conversion with a framework reconstruction. Figure 2e shows that the N_2_ adsorption–desorption isotherms of solid core/porous shell SiO_2_ particles. As expected, they exhibit typical type-IV features of absorbents with a H2 hysteresis. According to the Brunauer–Emmett–Teller method, the specific surface area was estimated to be 450 m^2^ g^−1^. The pore size distribution in the SiO_2_ particles calculated by the Barrett–Joyner–Halenda method from adsorption branch shows that the pores are uniform with an average of 3.2 nm (Supplementary Fig. 5a). After converted to hp-SiNSs, the absorption–desorption hysteresis transits from H2 to H3 (Fig. 2f) and thus the pore size distribution becomes broader (Supplementary Fig. 5b)^25^. Such changes occur owing to the reorganization of building blocks during the transformation, as reported for Si thin film by Tolbert and colleagues^26^. Consistently, Barrett–Joyner–Halenda calculation shows the pore size ranges from two to tens of nanometres. A high Brunauer–Emmett–Teller surface area of 550 m^2^ g^−1^ was achieved, which was among the largest surface area ever reported^27^,^28^. The surface area of solid core is much smaller than that of porous shell in the starting solid core/porous shell SiO_2_ particles. In view of the similar density of silica (2.2 g cm^−3^) and silicon (2.3 g cm^−3^), the increase of surface area after conversion can be mainly ascribed to the removal of the solid core.

### Electrochemical performance

**Figure 3:**
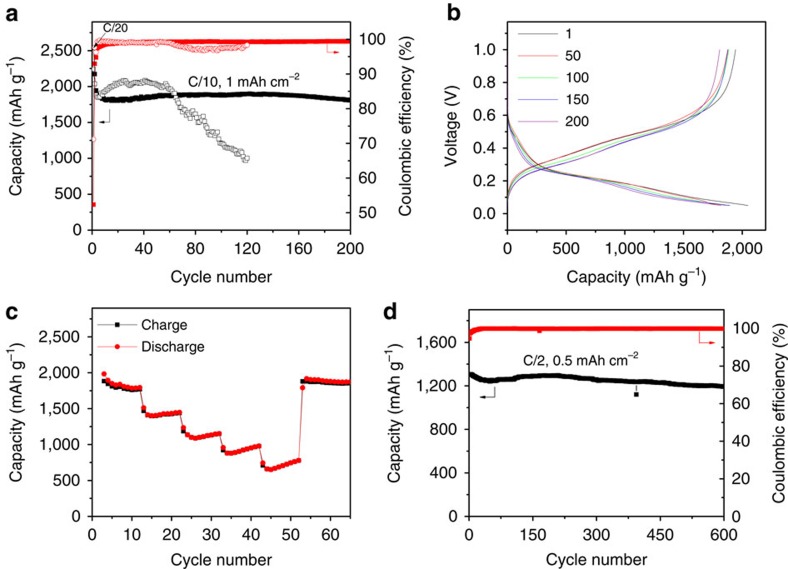
Electrochemical performance of the hp-SiNSs. (**a**) Lithiation capacity and Coulombic efficiency of the hp-SiNSs and commercial solid 100 nm Si particle electrode cycled between 1 V and 0.05 V at 0.1C with the loading of 1 mAh cm^−2^. (**b**) Galvanostatic charge–discharge profiles during cycling. (**c**) Lithiation capacity of the hp-SiNS electrode at various rates from 0.1C to 2C. (**d**) Lithiation capacity and Coulombic efficiency of the hp-SiNS electrode cycled between 1 V and 0.05 V at 0.1C with the loading of 0.5 mAh cm^−2^.

The hp-SiNSs show remarkable performance as an anode for LIBs, as described below. The charge/discharge capacity versus cycle number is presented in Fig. 3a. The reversible specific capacity reaches 1,850 mAh g^−1^ at 0.1C (1C=3.6 A g^−1^). Such a high specific capacity value indicates that most of Si is active owing to the high Li^+^ accessibility of the hierarchically porous structures. After the two-cycle formation step at C/20, the capacity is maintained above 1,800 mAh g^−1^ after 200 cycles, demonstrating excellent capacity retention of the hp-SiNSs. The Coulombic efficiency increases from 52% at the first cycle to above 99.0% after twentieth cycle. The irreversible capacity loss at the first cycle can be compensated by prelithiation through either chemical or electrochemical methods or by using stabilized lithium metal powder^29^. In the following cycles, a stable SEI is formed and the Coulombic efficiency reached up to 99.4%. Under similar conditions, the commercial Si nanoparticles show fast capacity decay, from 2,000 mAh g^−1^ at initial cycles to <1,000 mAh g^−1^ after 100 cycles (Fig. 3a). Such comparative results indicate that the enhanced cyclability of the hp-SiNSs mainly stems from their unique porous structure rather than the binder or the conductive additives. In consistent with cyclic voltammetry results (Supplementary Fig. 6), the voltage profile in Fig. 3b shows the electrochemical behaviour of amorphous Si. The rate performance tests were carried out at various rates from C/10 to 2C, as shown in Fig. 3c. The discharge capacities of 1,850, 1,430, 1,125, 920 and 700 mAh g^−1^ were obtained at the rates of C/10, C/5, C/2, C and 2C, respectively. The cyclability can be improved to 600 cycles at a rate of C/2 when the loading is decreased from 1 to 0.5 mAh cm^−2^ (Fig. 3d). The average Coulombic efficiency is as high as 99.91%. It is worthy to point out that the current electrodes show moderate improvement in volumetric capacity (∼760 mAh cm^−3^) against graphitic anodes (∼620 mAh cm^−3^)^13^. As compared with the currently available Si materials, our hp-SiNSs are among the most promising candidates as anode for high-performance LIBs in light of both cycling performance and rate capability^7^,^8^,^9^,^10^,^11^,^12^.

### *In situ* TEM observation of lithiation/delithiation

**Figure 4:**
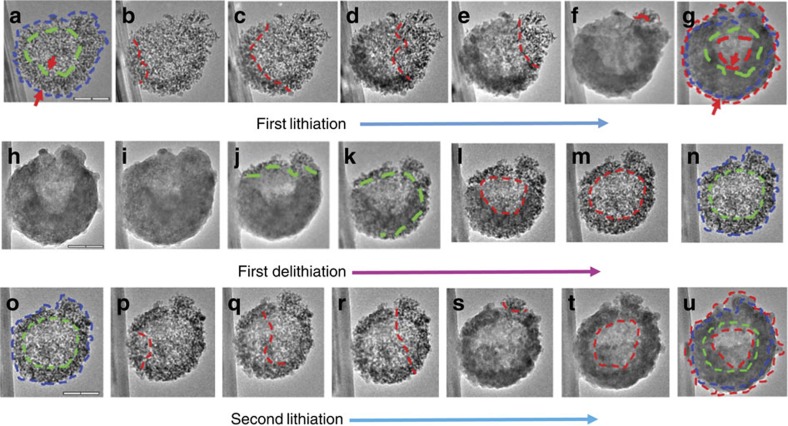
*In situ* TEM characterization of the lithiation/delithiation behavior of a hp-SiNS. *In situ* TEM images of the first lithiation process at (**a**) 0 s, (**b**) 889 s, (**c**) 1,364 s, (**d**) 2,000 s, (**e**) 2,708 s, (**f**) 3,946 s and (**g**) 16,813 s; the delithiation process at (**h**) 0 s, (**i**) 6,000 s, (**j**) 6,420 s, (**k**) 6,960 s, (**l**) 7,154 s, (**m**) 7,860 s and (**n**) 9,060 s; the second lithiation process at (**o**) 0 s, (**p**) 40 s, (**q**) 480 s, (**r**) 1,104 s, (**s**) 2,065 s, (**t**) 2,840 s and (**u**) 5,694 s. (The hollow pore is circled in green and the total size is circled in blue in panels **a** and **o** the red lines in panels **b**–**f** shows the interface between the lithiated and unreacted regions; in panel **g** both the hollow pore and the total size are circled in red and the red arrows indicate the thickness of the shell in panels **a**,**g**. The green dashed lines indicate the interface between delithiated and remaining regions in panels **j**,**k**, the hollow pore is circled in red in panels **l**,**m** and the hollow pore is circled in green and total sphere is circled in blue in panels **n**,**o**. The red lines in panels **p**–**s** divide the lithiated and unlithiated regions, the hollow core is circled in red in panel **u** and the hollow core and total size of the Si sphere are circled by two red circles. The green and blue circles in panels **a**,**o** are overlaid on top of panels **g**,**u**, respectively). Scale bar, 200 nm (**a**,**h**,**o**).

The remarkable performance of this material is attributed to its unique volume accommodation mechanism, as revealed by the *in situ* TEM imaging under a dynamic operating condition of the half-cell nano battery. Figure 4a–g shows the first lithiation process of a hp-SiNS (Supplementary Movie 1). The projected area of the pristine hp-SiNS at the initial state is 182,867 nm^2^, as circled in blue in Fig. 4a. On bringing the Li source to the close contract of the sphere, Li^+^ ions diffuse quickly from the contact point via surface diffusion into the hp-SiNS through a wave-propagation-like motion, as shown in Fig. 4b–f. As lithiation proceeded, the mesopores in the shell shrunk and gradually disappeared, and the shell thickness of the hp-SiNS increased from 113 nm at 0 s to 189 nm at the end of the lithiation at 16,813 s. However, the increase in the outer diameter of the hp-SiNS was insignificant since the lithiation-induced volume increase was largely accommodated by the inward expansion of the hp-SiNS to fill the hollow pore. The total projected area of the fully lithiated hp-SiNS was increased to ∼233,000 nm^2^. The total area increase was 27% after full lithiation based on TEM projection images. Assuming isotropic volume expansion of the amorphous Si, the total volume expansion was calculated to be around 44% (which is around 1.27^3/2^–1). Considering the 300% volume expansion for solid Si anodes^30^, the porosity of the original hp-SiNS is estimated to be 0.64. In addition, the wave-propagation lithiation in our samples is in distinct contrast to the core-shell lithiation of the solid Si nanostructures, as reported in previous studies^31^,^32^. This different lithiation kinetics is most likely due to the extremely high surface areas in our samples. The core-shell lithiation in solid Si structures tends to generate large hoop tension at the lithiated shell, leading to surface fracture or pulverization of the Si structures^31^,^32^,^33^,^34^,^35^. Furthermore, the compressive stress at the reaction front retards further lithiation, limiting the rate performance and loading efficiency of the Si anodes^30^,^31^,^36^. The ultrafast diffusion through pore surfaces and wave-propagation-like lithiation exhibited by our hp-SiNSs simultaneously enhance the lithiation rate and alleviate the lithiation-induced stress, thereby giving rise to better rate performance and longer cycle life of the Si anodes.

Figure 4h–n shows the delithiation process of the hp-SiNS (Supplementary Movie 2). The starting state of the fully lithiated hp-SiNS is the same as Fig. 4g, but rotated to a different orientation. As shown in Fig. 4j–k, delithiation proceeded by the similar wave-propagation-like motion as seen in lithiation and the delithiated regions recovered to the original porous structure, where the green dashed lines mark the delithiation front. The hollow core expanded gradually as delithiation continued, as marked by the red circles in Fig. 4l,m. The fully delithiated state in Fig. 4n exhibited the same hollow center/porous shell structure as the pristine hp-SiNS in Fig. 4a. Therefore, the lithiation/delithiation processes of the hp-SiNS are highly reversible. In addition, the Si nanoparticles that constitute the hp-SiNS returned to the similar size and morphology. The total area of the fully delithiated state (around 176,000 nm^2^), as circled by blue dashed line in Fig. 4n, is similar to that in the initial state shown in Fig. 4a, while the measured area of the hollow core circled by green dashed line is of 70,872 nm^2^. Figure 4o–u show that the morphological and structural evolutions of the hp-SiNS during the second lithiation (Supplementary Movie 3) are very similar to the first lithiation. The highly reversible Li-breathing morphology during electrochemical cycling in our hp-SiNSs enables long cycle life of the Si anodes.

### Chemomechanical modelling

To further appreciate the inward breathing during lithiation/delithiation cycles of the hp-SiNS, we extend a recently developed chemomechanical model^37^,^38^ to simulate the concurrent processes of phase transformation, stress generation and morphological evolution of the three-dimensional hp-SiNS (Supplementary Note 1 and Supplementary Fig. 11). In the model, the hp-SiNS is simulated as an elasto-plastic material. The total strain *ɛ*_*ij*_ is composed of three parts, 
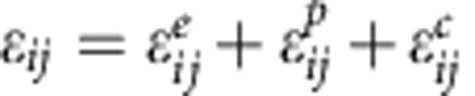
, where 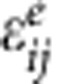 is the elastic strain, 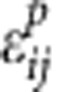 is the plastic strain and 
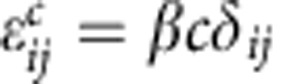
 is the chemical strain. Here *c* is the local Li concentration that varies from 0 (representing the unlithiated phase) and 1 (representing the fully lithiated phase), *β* is the expansion coefficient. Both the Young's modulus and yield strength of the hp-SiNS are dependent on the Li concentration. The Young's modulus of porous Si can be estimated by 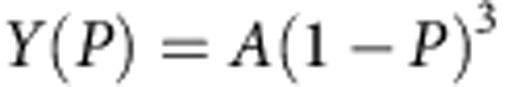, where *A*=169 GPa (ref. 39) is a constant and *P* is the porosity^40^. For *P*=0.64, the Young's modulus of is ∼7.9 GPa. The fully lithiated Si phase possesses a Young's modulus of ∼40 GPa (refs 41, 42, 43). We adopt a yield stress of 0.5 GPa for both the unlithiated porous Si and the fully lithiated Si. The relatively low yield stress of the unlithiated porous Si is in accordance to the ease of generating plastic flow observed in the experiments. Both the Young's modulus and the yield strength are set to be nonlinearly dependent on Li concentration. Li insertion into porous Si induces two competing effects: a weakening effect on the Si–Si bonds in the presence of Li and a strengthening effect due to Li-insertion-induced volume expansion and subsequent pore shrinkage^44^,^45^. It can be anticipated that both the Young's modulus and yield strength first decrease as the Li concentration increases since the weakening effect of Li dominates. This trend reverses beyond a critical Li concentration at which the strengthening effect becomes dominant. With given materials properties of the pristine porous structure and the fully lithiated phase, we nonlinearly interpolate the Young's modulus and the yield stress, with the lowest values set at a Li concentration of 72% (corresponding to the Li_2.6_Si phase). On the basis of the experimental observations, we set the surface diffusivity of Li to be two orders of magnitude larger than its bulk diffusivity. The diffusion equations and the mechanics equilibrium equations are solved simultaneously to obtain the deformation morphology and the Li concentration profile at any given lithiation/delithiation state.

**Figure 5:**
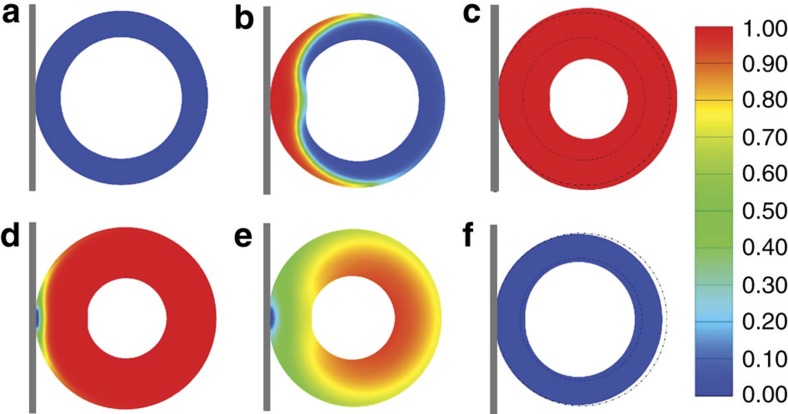
Chemomechanical modelling of the lithiation/delithiation processes of a hp-SiNS. For better visualizing the processes, only a cross-section is shown. Colours denote the Li concentration, with red being fully lithiated and blue unlithiated. (**a**) The Li source, the rigid plate on the left, is brought to contact with the hp-SiNS. (**b**) Li diffuses into the hp-SiNS in a wave-propagation manner. The newly lithiated product at the reaction front pushes inward more than outward because of the lower stiffness of the inner unlithiated porous layer than that of the outer lithiated layer. (**c**) On fully lithiation, the Si sphere is slightly distorted, and the inward volume expansion is significantly larger than the outward volume expansion. (**d**–**f**) Delithiation also proceeds by a wave-propagation-like motion. Inner materials are dynamically pulled to the outer surface of the Si sphere due to the delithiation-induced tensile stress.

Figure 5 shows the snapshots of cross-section morphologies for both the lithiation and delithiation processes simulated by our chemomechanical modelling. Li source is placed at the contact point between a rigid plate and a hollow porous sphere. Lithiation proceeds by a wave-propagation manner from the Li source (Fig. 5b and Supplementary Movie 4), successfully mimicking the lithiation kinetics observed in the experiments. For our continuum model to take into account of the porosity of the Si sphere, the mesoporous structure undergoes phase transformation but no volume expansion until lithiation to Li_2.6_Si. Further lithiation to Li_3.75_Si (the fully lithiated stage) gives rise to the apparent 44% volume expansion. At the reaction front, high incompatible strain is generated. To relax the high strain energy, the newly generated volume by lithiation pushes against both the lithiated outer layer and unlithiated porous layer^37^,^38^,^46^,^47^. The outward pushing effect generates hoop tension in the lithiated layer, while the inward pushing effect generates compression in the unlithiated porous layer. Owing to the lower stiffness of the inner unlithiated porous layer than that of the outer lithiated layer, the mechanical resistance for the reaction front to push inward is much smaller than outward, resulting in significantly stronger inward than outward expansion, as shown in Fig. 5c in the fully lithiated state, where the dashed lines mark the initial inner and outer surfaces in the original configuration (Fig. 5a). The fully lithiated configuration is slightly distorted, owing to the asymmetric lithiation starting from a point source. It should be noted that the inward volume expansion is precisely opposite to the volume expansion accommodation mechanism during the lithiation of a solid Si structure, where the unlithiated core is much stiffer than the lithiated shell, leading to negligible inward expansion but significant outward expansion^34^,^46^,^48^,^49^. Such core-shell lithiation has been widely known to generate large hoop tension in the surface layer of the lithiated Si, causing surface fracture^34^,^35^,^46^. During delithiation, Li is drawn back to the plate that acts as a Li sink, creating a Li concentration gradient. Our simulations show that delithiation also proceeds by the wave-propagation-like motion in Fig. 5d–f and Supplementary Movie 5. Owing to a lower Li concentration and hence a larger shrinkage in the outermost region, tensile stress is generated in the regions with higher Li concentration during delithiation. The inner materials are thus dynamically pulled towards the outer region of the hollow porous sphere, thereby almost completely recovering the pristine hollow porous structure.

## Discussion

We have developed a novel chemical transformation method for the synthesis of hierarchically porous Si. Our starting material consists of submicron solid core/mesoporous shell SiO_2_ spheres, which can be as an assembly of submicron and nanometre building blocks. The fast surface reaction in the nanoporous shell and slow bulk reaction in the solid SiO_2_ core lead to porous Si/solid SiO_2_ core on magnesiothermal reduction. Further acid etching of the solid SiO_2_ core gives rise to the hp-SiNSs. In contrast, conventional transformation is commonly applied to nanostructure building blocks at a single length scale^50^, generally leading to complete conversion of chemical composition. Our chemical transformation method innovatively exploits the nanostructure size-dependent reaction kinetics, which opens a new avenue for the synthesis of hierarchically structures materials.

Our *in situ* TEM characterization has demonstrated that the hp-SiNS features inward volume expansion and contraction on lithiation and delithiation, in distinct contrast to the huge outward volume changes of solid Si. Our chemomechanical modelling reveals that both the mesoporous shell and hollow internal void are indispensable for the inward expansion and contraction during lithiation and delithiation. The inward lithium breathing facilitates stable SEI formation, enhances the capacity retention and cycling life as compared with previously reported hollow nanospheres^8^,^9^, or porous Si particles^12^. Those nanostructures had either dense shell or not well-defined pores, or only uniformly sized pores, and consequently exhibited inferior capacity retention and limited cycling life. The hp-SiNSs have also shown improved capacity retention and Coulombic efficiency relative to other nanostructured Si materials, including nanoparticles^17^, nano-Si/carbon black composite^7^, nanowires^10^ and nanotubes^11^. Overall, the hp-SiNSs have demonstrated potential applications as high-performance anodes. For practical usage, it is critically important in future work to increase areal loading to pair with various cathodes through engineering of electrodes and utilization of improved electrolytes.

In conclusion, the hp-SiNSs exhibit ultrahigh Li diffusion and reversible inward volume expansion/contraction, which overcome the technical barriers for using solid Si as high-capacity anodes. The design and synthesis of such hp-SiNSs constitute a generic strategy to simultaneously enhance rate performance and structural stability of high-capacity electrode materials. The size-dependent reaction kinetics provides exceptional potential in rational design of hierarchically nanostructured materials and can be broadly applied to other materials such as SiC and Si_3_N_4_.

## Methods

### Synthesis of solid core/mesoporous shell SiO_2_ spheres

The solid core/mesoporous shell SiO_2_ spheres produced by simultaneous hydrolysation and condensation of tetraethoxysilane and octadecyltrimethoxysilane followed by removal of organic species. Absolute ethanol (117 g, 2.54 mol), deionised water (20 g, 1.12 mol) and aqueous ammonia (32 wt%, 5.64 g and 0.34 mol) were mixed in a 250 ml flask. After heating to 30 °C, tetraethyl orthosilicate (11.2 g, 0.052 mol) was added rapidly under stirring. After 1 h, a mixture of tetraethyl orthosilicate (9.34 g, 0.044 mol) and n-octadecyltrimethoxysilane (3.536 g, 0.00944, mol) was added drop by drop over a period of 20 min. After the mixture was added, the solution was kept at ambient temperature for 12 h. The resulting white powder was obtained by centrifuge and then calcined in air for a period of 6 h at 550 °C (1 °C min^−1^).

### Synthesis of hp-SiNSs

SiO_2_ powers (0.5 g) were dispersed in ethanol and cast onto a ceramic plate that was 1 cm away from 1 g of Mg powder layer dispersed at the bottom of a standard steal chamber. After the chamber was transferred into the tubular furnace, temperature was ramped to 680 °C at the rate of 10 °C and soaked for 2 h under Ar. The product was washed with dilute mixed acid (acetic acid: hydrochloric acid=4:1), followed by etching in hydrofluoric acid solution. Finally a Si brown powder was obtained under vacuum drying at room temperature and stored in glove box.

### Electrode fabrication, cell assembly and testing

The hp-SiNSs, conductive poly (9,9-dioctylfluorene-co-fluorenone-co-methyl-benzoic ester), and XG graphene nanoplatelets (Supplementary Fig. 7) with a weight ratio of 70:20:10 were dispersed in tetrahydrofuran. The slurry was cast on a copper foil and dried at room temperature. The electrode loading (including binder and carbon) is about 0.8 mg cm^2^. Later the electrodes were punched into a circular disc with a diameter of 0.5″ and further treated by ramping the temperature from room temperature to 500 °C at the rate of 10 °C and soaking for1 h under Ar. The heat treatment for the electrode can improve the performance by the removal of the CHx species (Supplementary Fig. 8). After heat treatment the Si content increases to 76 wt% based on the mass change. The silicon loading is about 1 mAh cm^−2^. For comparison, the commercial solid 100 nm Si nanoparticles were used to fabricate benchmark electrodes under the similar conditions (Supplementary Fig. 9). The coin cells, composed of a Si electrode, a microporous polyethylene separator, a lithium counter electrode were assembled in an argon-filled glove box. The electrolyte was a 1.0 M LiPF_6_ solution in ethylene carbonate/diethyl carbonate (2/1 vol%) with 10 wt% fluorinated ethylene carbonate as an additive. The galvanostatic charge and discharge measurements were taken on a Maccor testing system. The Cyclic voltammetry was obtained using BioLogic workstation in a home-made three-electrode configure.

### Characterizations

TEM, scanning TEM images and the energy dispersive X-ray spectroscopy are taken on JEOL JEM 2,100 F at 200 kV. The X- ray diffraction patterns are obtained on Bruker D8 Advance with Cu Kα radiation (*λ*=1.5418 Å). *In situ* TEM is carried out on a FEI Titan microscope at 300 kV. To elucidate the structural advantage of the hp-SiNSs on electrochemical performance, *in situ* observation of the structural changes of the hp-SiNSs is conducted by assembling a nano battery inside of the TEM column as shown in Supplementary Fig. 10. A hp-SiNS is loaded onto a Si nanowire (SiNW). The SiNW is grown on Si substrate, which is further connected to the Au rod using conductive epoxy. During the *in situ* lithiation, the SiNW is connected to the Li/Li_2_O end. With external bias applied, Li ions diffuse through the Li_2_O solid electrolyte and react with the SiNW first. In the same time, the Li ions diffuse quickly along the SiNW to the hp-SiNS and react with it.

## Additional information

**How to cite this article:** Xiao, Q. *et al.* Inward lithium-ion breathing of hierarchically porous silicon anodes. *Nat. Commun.* 6:8844 doi: 10.1038/ncomms9844 (2015).

## Supplementary Material

Supplementary Information Supplementary Figures1-11, Supplementary Note 1 and Supplementary References

Supplementary Movie 1 Lithiation of a hp-SiNS for the first cycle. Note that the real time of the whole movie is 16813 s, but compressed to 35 s for easy view and comparison.

Supplementary Movie 2 Delithiation of a hp-SiNS for the first cycle.

Supplementary Movie 3 Lithiation of a hp-SiNS for the second cycle.

Supplementary Movie 4 Chemomechanical modeling of the lithiation of hp-SiNS

Supplementary Movie 5 Chemomechanical modeling of the delithiation of hp-SiNS
