# Supplementary material for: Inward lithium-ion breathing of hierarchically porous silicon anodes
Source: Nat Commun. 2015 Nov 5;6:8844. doi: 10.1038/ncomms9844 (PMC4667626; doi:10.1038/ncomms9844)
Supplement: Supplementary Information — Supplementary Figures1-11, Supplementary Note 1 and Supplementary References [file ncomms9844-s1.pdf]

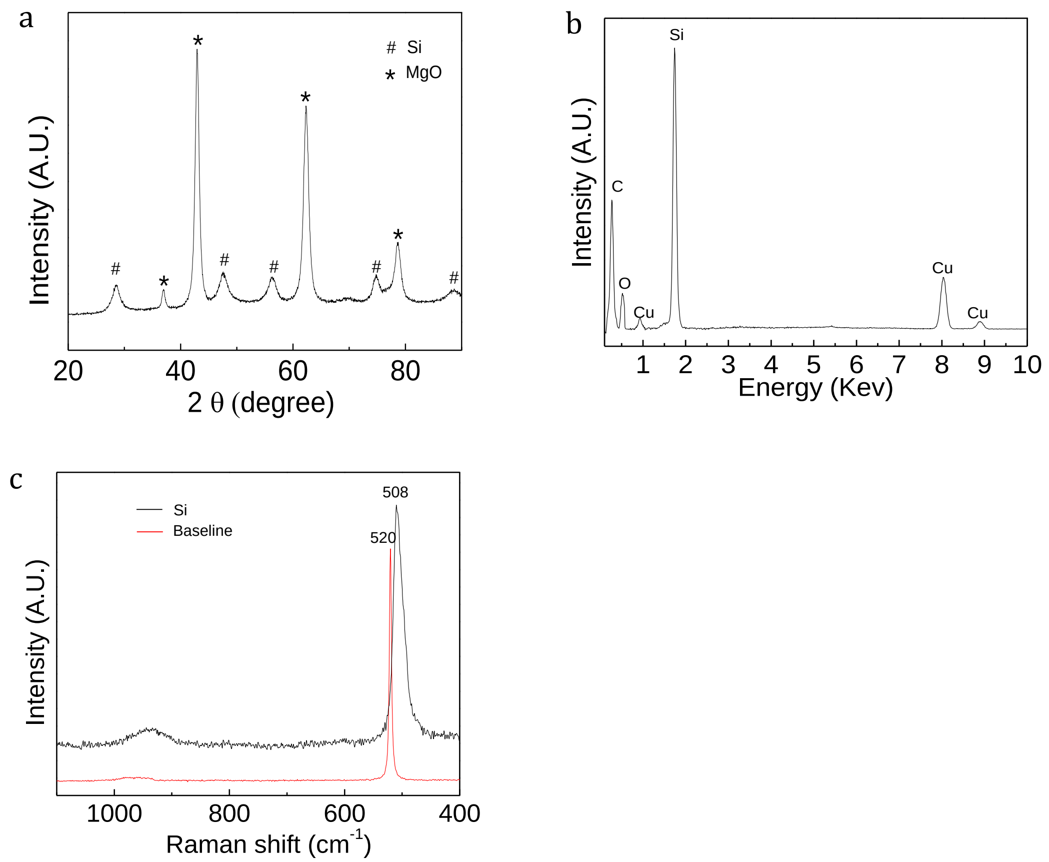

Supplementary Figure 1 | Phase and chemical analysis of the product. (a) XRD patterns of the intermediate product before acid treatment. (b) Energy dispersive X-ray spectroscopy (EDX) analysis (Cu signal from the TEM copper grid) and (c) Raman spectra of the hp-SiNSs obtained by acid treatment of the intermediate product.

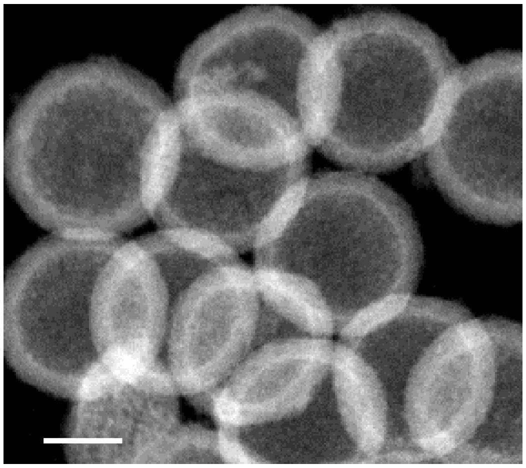

Supplementary Figure 2 | Scanning transmission electron microscopic (STEM) image of hp-SiNSs. The scale bar is 200 nm.

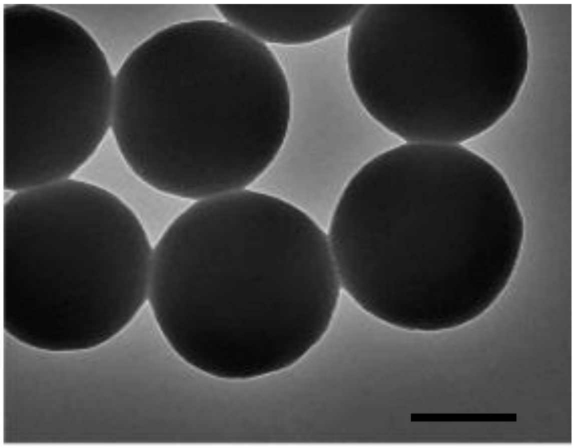

Supplementary Figure 3 | Transmission electron microscope (TEM) image of solid silica spheres. The scale bar is 200 nm.

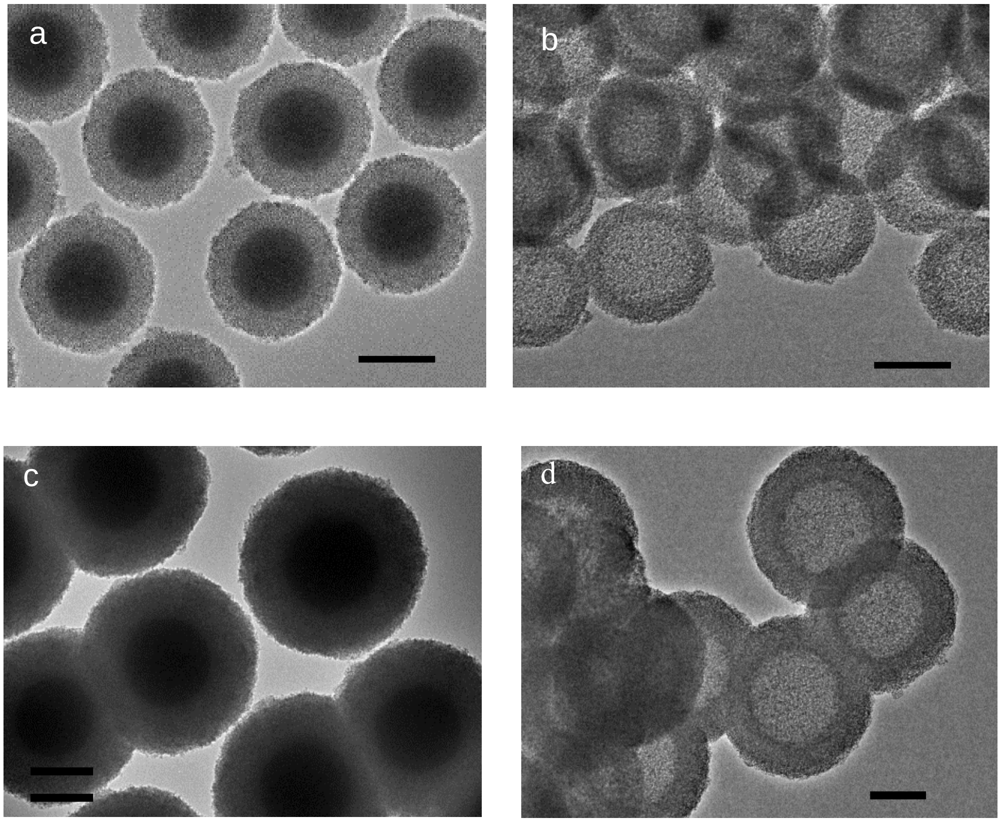

Supplementary Figure 4 | hp-SiNSs with tunable diameter and shell thickness. (**a**) TEM image of solid core/mesoporous shell (SCMS) SiO_2 particles with a diameter of 350 nm and shell of 75 nm, (**b**) TEM image of the corresponding hp-SiNSs. (**c**) TEM image of solid core/mesoporous shell (SCMS) SiO_2 particles with a diameter of 590 nm and shell thickness of 150 nm, (**d**) TEM image of the corresponding hp-SiNSs. The scale bar is 200 nm.

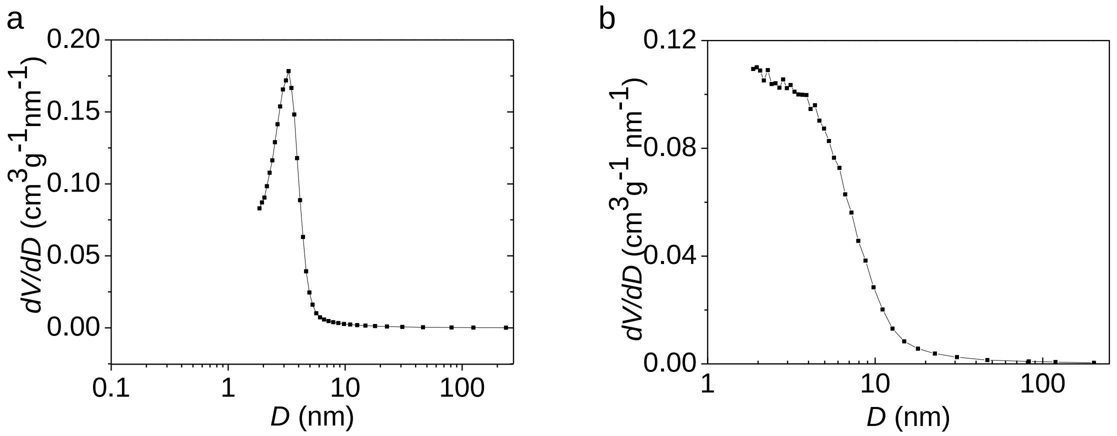

Supplementary Figure 5 | The pore size distribution in (a) solid core/porous shell SiO_2 particles and (b) hp-SiNSs, calculated by the Barrett-Joynes-Halenda (BJH).

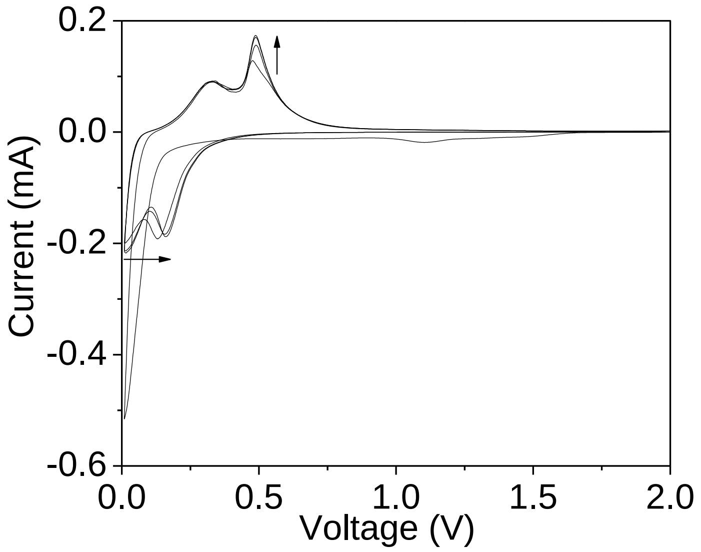

Supplementary Figure 6 | Cyclic voltammetry of the Si particle electrode in the potential window of 0.01 to 2 V versus Li/Li+ collected at the rate of 0.020 mV s^{-1}. Note the arrows indicate the shift with cycling.

a

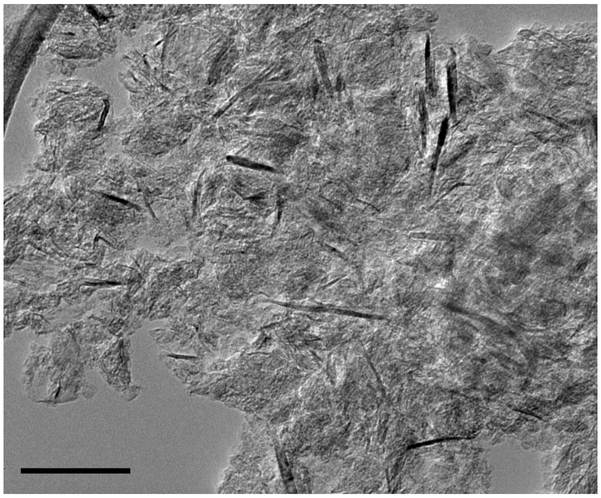

b

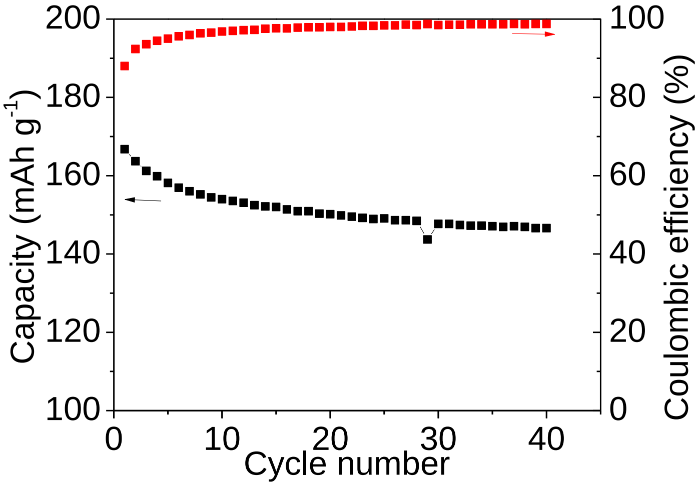

Supplementary Figure 7 | (a) Transmission electron microscope (TEM) image of XG graphene nanoplatelets. (b) Lithiation/delithiation capacity of the XG graphene nanoplatelet electrode cycled between 1 V and 0.05 V at 0.1C at 25 °C. The scale bar is 100 nm.

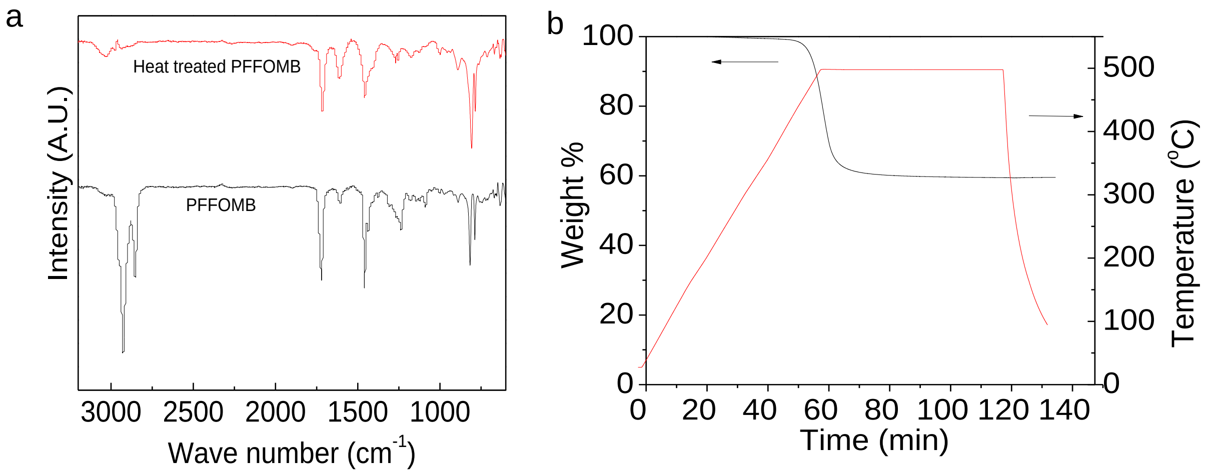

Supplementary Figure 8 | (a) FTIR spectra of Poly (9, 9-dioctylfluorene-co-fluorenone-co-methyl-benzoic ester) (PFFOMB) before and after heat treatment, namely, ramping to 500 °C from room temperature in 1 h and soaking for another 1 h under Ar. (b) Thermogravimetric analysis (TGA) of PFFOMB by ramping to 500 °C from room temperature in 1 h and soaking for another 1 h under Ar.

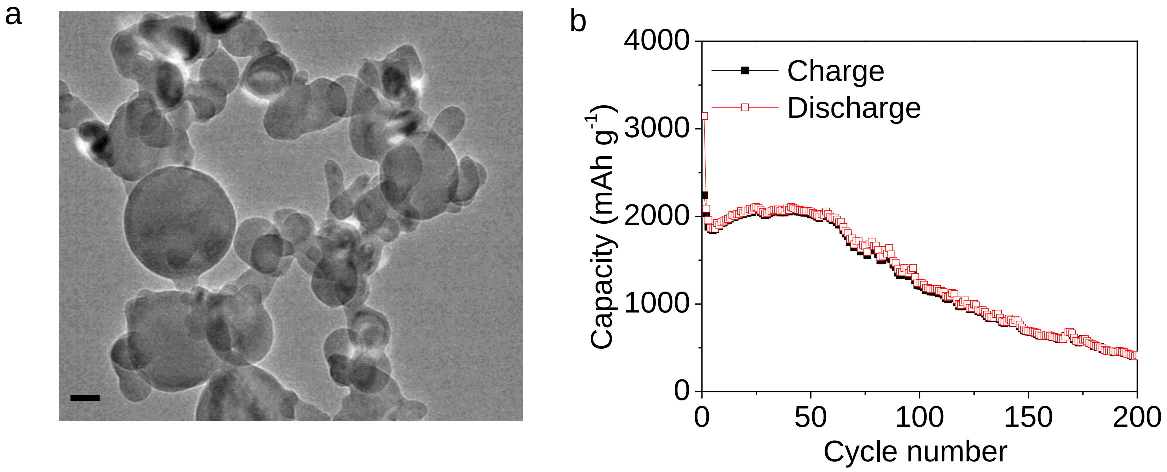

Supplementary Figure 9 | (a) Transmission electron microscope (TEM) image of commercial Si nanoparticles. (b) Lithiation/delithiation capacity of the Si nanoparticle electrode cycled between 1 V and 0.05 V at 0.1C at 25 °C. The scale bar is 50 nm.

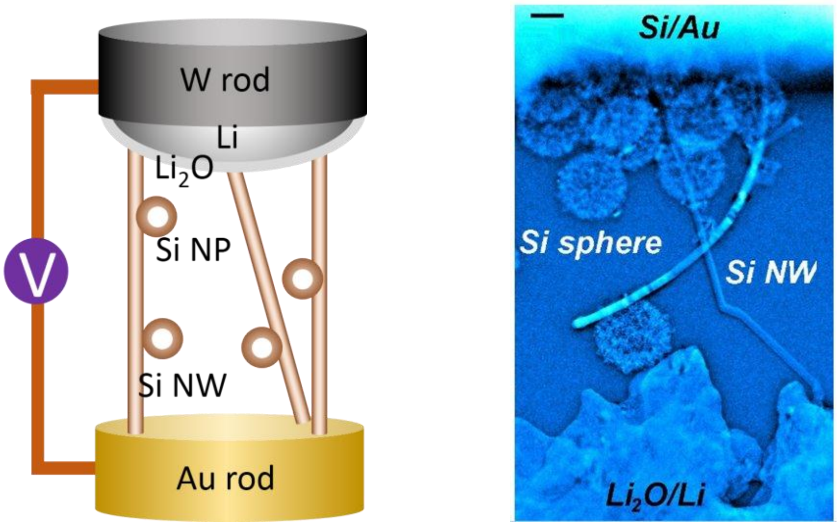

Supplementary Figure 10 | Setup of nanobattery for in-situ TEM observation. The scale bar is 200 nm.

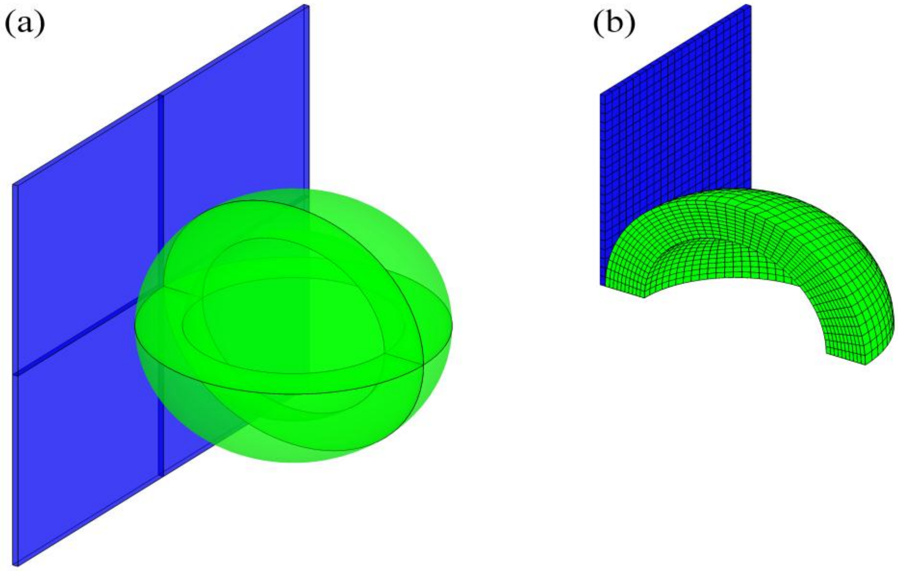

Supplementary Figure 11 | Simulation setup of a hp-SiNS under lithiation. (a) Solid model used in the simulation, consisting of a rigid substrate representing the counter electrode and a single hp-SiNS. The Li source is directly set at the point on the outer surface of the hp-SiNS which contacts with the substrate. (b) Finite element mesh adopted to discretize the solid model.

Supplementary Note 1

Chemo-mechanical modeling for hp-SiNS

To simulate the lithiation process in hp-SiNS, a recently developed chemo-mechanical model is adopted here[1, 2]. In the model, the Li diffusion in hp-SiNSs is surrogated by heat conduction, and the normalized Li concentration c is regarded as temperature in heat conduction. By solving a coupled boundary value problem:

$$\begin{cases} \nabla \cdot \boldsymbol{\sigma} = 0 \\ \dfrac{\partial c}{\partial t} = \nabla \cdot \left(D \nabla c \right) \end{cases} \quad \text{in } V \tag{1}$$

with pertinent boundary and initial conditions, the dynamic evolution of Li concentration, morphological change, and stress generation in lithiated hp-SiNSs can be obtained. In Eq.(1), $\boldsymbol{\sigma}$ is the Cauchy stress tensor, ∇ represents the vector differential operator with respect to spatial coordinates, D is the Li diffusivity, which is considered to be concentration dependent only in this study, V is the volume of the hp-SiNSs. Using the ABAQUS/Standard implicit coupled temperature-displacement procedure in the finite element package ABAQUS[3], the coupled chemo-mechanical model is solved and Li concentration and stress-strain fields in the hp-SiNSs are updated incrementally. Meanwhile, the user subroutine UMATHT is used to interface with ABAQUS to dynamically update the diffusivities based on the instantaneous Li profile.

As shown in Supplementary Figure 11 (a), a hp-SiNS is attached to a rigid substrate that represents the counter electrode. Surface-to-surface contact interaction is applied between the surface of the substrate and the outer surface of the hp-SiNS. In order to simplify the model and ignore the Li diffusion across the contact surface, the Li source is directly set at the point on the outer surface of the hp-SiNS which contacts with the substrate ($c = 1$). In addition, the symmetry boundary condition is used to reduce the computational cost of the model. Therefore, only a quarter of the solid model is considered in the simulation, and discretized by 8-node brick element, as shown in Supplementary Figure 11 (b).

Supplementary References

1. Yang H. *et al.* Orientation-dependent interfacial mobility governs the anisotropic swelling in lithiated silicon nanowires. *Nano Lett.* **12,** 1953-1958 (2012).
2. Yang, H. *et al.* A chemo-mechanical model of lithiation in silicon *J. Mech. Phys. Solids* **70,** 349-361 (2014).
3. Dassault Systèmes. *Abaqus analysis user's manual V. 6.10* (Dassault Systèmes Simulia Corp., 2010).
